# Arterial-related craniospinal physiological compliance assessed by phase-contrast MRI and lumbar infusion test

**DOI:** 10.1093/braincomms/fcag126

**Published:** 2026-04-08

**Authors:** Kimi Owashi, Cyrille Capel, Olivier Balédent

**Affiliations:** Medical Image Processing Department, CHU Amiens-Picardie, Amiens 80000, France; CHIMERE INSERM UA21, Jules Verne University of Picardy, Amiens 80000, France; CHIMERE INSERM UA21, Jules Verne University of Picardy, Amiens 80000, France; Neurosurgery Department, CHU Amiens-Picardie, Amiens 80000, France; Medical Image Processing Department, CHU Amiens-Picardie, Amiens 80000, France; CHIMERE INSERM UA21, Jules Verne University of Picardy, Amiens 80000, France

**Keywords:** craniospinal compliance, phase-contrast MRI, lumbar infusion test, cerebral arterial flow, normal pressure hydrocephalus

## Abstract

Compartmental compliance is classically defined as the ratio between volume change and resulting pressure change. Nevertheless, the concept of craniospinal compliance is inherently complex, as it involves different physiological mechanisms that vary according to temporal and volumetric scales. During lumbar infusion tests, the craniospinal system is stressed artificially, whereas during a single cardiac cycle, it operates under dynamic physiological conditions. In this study, we propose a novel approach for estimating arterial-related craniospinal compliance under physiological conditions by combining data from phase-contrast MRI and lumbar infusion test. This physiological compliance is then compared with craniospinal compliance-related parameter derived from the infusion test. We retrospectively included 108 patients (73 ± 8 years; 77 men) suspected with normal pressure hydrocephalus. Each participant underwent MRI examination, followed by a lumbar infusion test, with a mean interval of 46 ± 43 days between procedures. Cerebral arterial flows were extracted from phase-contrast MRI to compute total cerebral arterial volume change over one cardiac cycle. Craniospinal pressure change over one cardiac cycle was derived from the amplitude of the average intracranial pressure pulse extracted from baseline recordings during the lumbar infusion test. Arterial-related craniospinal physiological compliance was calculated as the ratio of volume change to pressure change over a single cardiac cycle. In parallel, craniospinal infusion-derived compliance was calculated by dividing the infused volume (from infusion start to pressure plateau) by the corresponding average pressure change. Arterial-related compliances were significantly higher than infusion-based compliance (*P* < 0.001); however, moderate but significant positive correlations were observed between them (*R* = 0.46; *P* < 0.001). These findings indicate that the two craniospinal compliance estimates, although derived under different physiological and temporal conditions, are complementary. Both describe the behaviour of a single craniospinal system but capture distinct mechanisms of adaptation: the rapid, pulsatile adjustments to arterial inflow during the cardiac cycle and the slower compensatory responses to sustained volume loading during the infusion test. Our findings support the view that craniospinal compliance is not a fixed property but rather a dynamic parameter that depends on the physiological context and the specific question addressed. Recognizing this complexity may enhance diagnostic precision and inform better-targeted treatment strategies in patients with suspected normal pressure hydrocephalus.

## Teaser

Owashi *et al.* report that craniospinal compliance measured under physiological and infusion conditions differs significantly but remains moderately correlated. Both methods capture complementary aspects of a single craniospinal system, demonstrating that compliance is a dynamic, context-dependent property relevant to improving diagnosis and treatment of patients with normal pressure hydrocephalus.

## Graphical Abstract

**Graphical Abstract:**
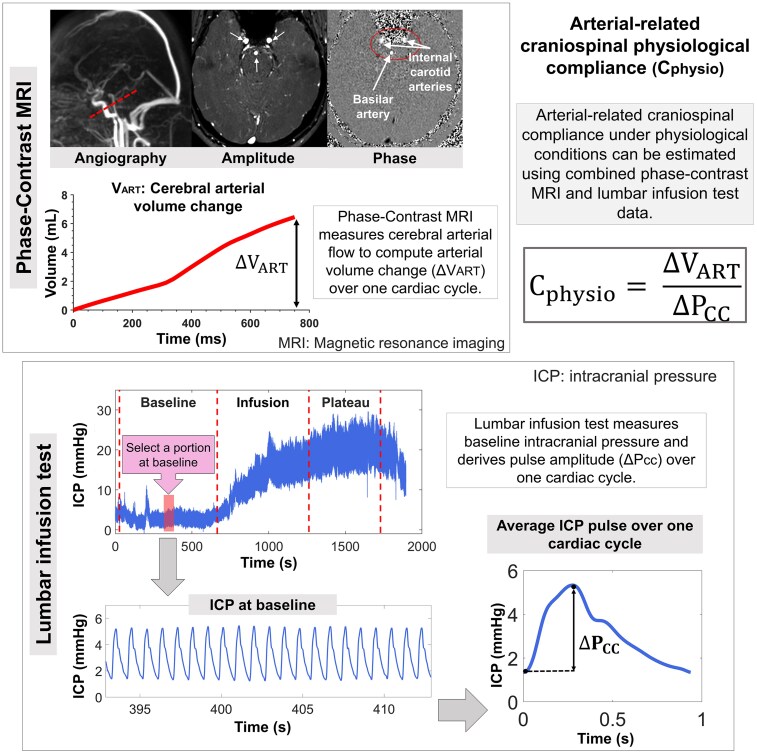

## Introduction

Compartmental compliance is simply defined as the volume change (ΔV) divided by the corresponding pressure change (ΔP) resulting from ΔV. It characterizes the ability of a system to accommodate volume changes with minimal pressure variation. A highly compliant system will exhibit only a small change in pressure following a volume increase, whereas a poorly compliant (or non-compliant) system will show a substantial pressure rise for the same volume change.

During the last decades, various studies have hypothesized reduced compliance in patients with normal pressure hydrocephalus (NPH), highlighting the importance of this parameter in disturbing the dynamics of the craniospinal system.^1-3^ These alterations are closely interrelated with ventricular dilation,^4^ altered CSF oscillations,^2,5^ and disturbances in intracranial pressure (ICP) dynamics,^6^ although the direction of these relationships is not definitively established and likely reflects a complex interplay rather than a simple cause–effect mechanism.

Often ambiguously referred to as intracranial, spinal, craniospinal, brain, or CSF compliance, this concept is frequently cited in both clinical practice and scientific literature. However, its precise definition, reliable measurement and even the appropriate terminology remain unclear—raising the question: are we all referring to the same physiological phenomenon? Several approaches have been proposed to estimate this compliance. Marmarou *et al.*^7^ introduced a technique in which the pressure response is recorded following the injection of a known volume into the CSF space, typically via a bolus injection. Okon *et al.*^8^ employed ultrasound-guided lumbar puncture, wherein CSF pressure is measured at each step of incremental CSF removal. Other approaches estimate compliance from ICP measurements obtained through infusion test combined with phase-contrast MRI (PC-MRI)^9^ or transcranial Doppler ultrasonography.^10^ PC-MRI has also been used in combination with non-invasive ICP estimation techniques.^11,12^ In addition, relative compliance indices have been derived solely from PC-MRI measurements,^13^ as well as from exclusively ICP-based recordings, including analyses of pulse amplitude in relation to mean ICP changes^14^ and variations in the P2/P1 ratio—where P1 and P2 represent characteristic peaks of the ICP pulse waveform.^15^

The infusion test, which enables dynamic assessment of ICP through the injection of physiological saline into the subarachnoid space, is considered a reference diagnostic tool for patients with NPH.^16-18^ This test estimates intracranial compliance using Marmarou’s electrical model of CSF dynamics,^7^ which assumes constant CSF secretion and absorption rates. However, the model does not account for transient changes in CSF production or reabsorption, or the volume changes induced by blood and CSF oscillations during the cardiac cycle. In contrast, PC-MRI is the only imaging technique capable of quantifying CSF and blood flow under physiological conditions throughout the cardiac cycle, thereby allowing estimation of the associated volume changes, although it does not provide information on the corresponding ICP changes.

Although both methods independently provide valuable information, their clinical interpretation remains challenging, particularly in patients with NPH, where the pathophysiology involves complex interactions between vascular and CSF dynamics. Existing diagnostic tests often rely on isolated markers, such as elevated CSF stroke volume at the aqueduct of Sylvius^19-21^ or high resistance to CSF outflow (*Rout*),^17,22^ which alone are insufficient to confirm NPH^23^ or predict shunt responsiveness.^24,25^

Using data from lumbar infusion test and PC-MRI, this study aims to estimate arterial-related craniospinal compliance under physiological conditions by accounting for the total cerebral arterial volume input to the craniospinal system and the resulting pressure change over the short time scale of the cardiac cycle. This physiological compliance is then compared with a craniospinal compliance-related parameter, estimated directly from ICP recordings obtained during the longer time scale of the lumbar infusion test and the corresponding infused volume. We hypothesized that these two measurements reflect distinct physiological mechanisms. By integrating these complementary techniques, we expect that the proposed physiological compliance marker could provide additional insights into NPH pathophysiology and ultimately enhance diagnostic accuracy and patient selection for shunt surgery.

## Materials and methods

### Study populations

We retrospectively included 108 patients suspected with NPH. The study population comprised 31 women and 77 men, with a mean age of 73 ± 8 years (range: 50–88 years). Each participant underwent an MRI examination, followed by a lumbar infusion test, with an interval of less than 5 months (mean: 46 ± 43 days) between these two procedures.

Among these patients, 29 (19 men and 10 women; mean age, 74 ± 6 years; range, 61–86 years) who underwent shunt surgery and had complete 6-month postoperative follow-up data were included in the NPH + subgroup. All these patients showed documented clinical improvement after placement of a pressure-regulated shunt (Polaris, Sophysa). Clinical status was assessed using the idiopathic NPH (iNPH) grading scale^26^ prior to shunt placement and again 6 months after shunting. Because cognitive assessment was incomplete, the iNPH grading scale was adapted to include only gait, balance and urinary disturbance evaluations. Clinical improvement was defined as an increase of at least 10% on the iNPH grading scale, assessing gait disturbances, living situation and urinary continence. Patients were selected for shunting by the neurosurgeon (C.C.) based on the combined analysis of tap-test, lumbar infusion test and/or PC-MRI results, together with clinical and radiological (Disproportionately Enlarged Subarachnoid space Hydrocephalus score^27^) findings.

All these patients came from the reversible dementia project research protocol (https://revertproject.org/). The study was conducted in accordance with the principles outlined in the Declaration of Helsinki and approved by the Ethics Review Committee (ID-RCB: 2021-A00240-41). All patients were informed of the objectives and procedures of the study. Prior to their participation in the study protocol, all subjects provided written informed consent.

### Phase-contrast MRI acquisition and processing

Patients were examined in the supine position, using a clinical 3 T MRI system (Philips Achieva; maximum gradient = 80 mT/m; slew rate = 120 mT m^−1^ ms^−1^) equipped with a 32-channel head coil.

**Figure 1:**
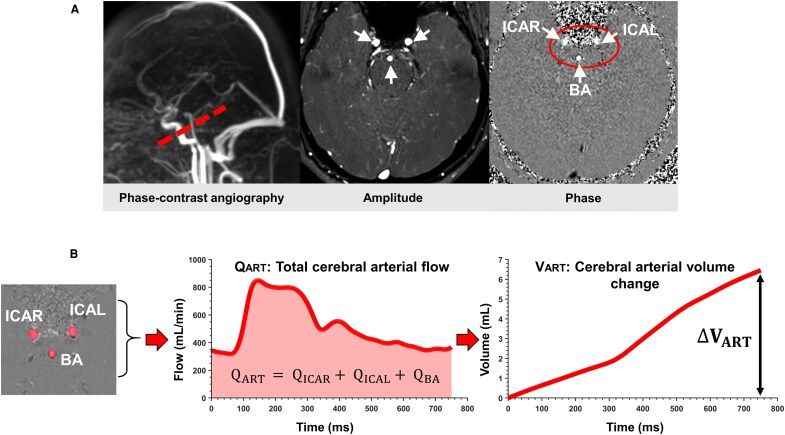
**Workflow for *ΔV_ART_* calculation.** (**A**) Sagittal 3D PC angiography was used to position the acquisition plane (below the Circle of Willis) perpendicular to the direction of arterial flow in major cerebral arteries. Labelled structures: ICAR, right internal carotid artery; ICAL, left internal carotid artery; BA, basilar artery. (**B**) PC imaging was used to quantify total cerebral arterial flow (Q_ART_). The cerebral arterial volume change curve (V_ART_) throughout the cardiac cycle was calculated by integrating the Q_ART_ over time. The amplitude of V_ART_ curve was taken as *ΔV_ART_*.

Sagittal 3D phase-contrast angiography [echo time (TE)/ repetition time (TR) = 3 ms/5 ms; field of view (FOV) = 350 (foot-head) 350 (anterior-posterior) 350 (right-left) mm^3^; spatial resolution = 1.5 × 1.5 × 1.5 mm^3^; flip angle = 12°] was used as a reference to position the acquisition plane (below the Circle of Willis) for cerebral arterial flow measurements (Fig. 1A). The measurements included three arteries: the left and right internal carotid arteries (ICAL, ICAR) and the basilar artery (BA).

The acquisition parameters for arterial PC-MRI were as follows: velocity encoding (VENC) = 600–800 mm/s; FOV = 142 × 142 mm^2^; acquisition spatial resolution = 1 × 1 mm^2^; thickness = 3 mm; flip angle = 30°; sensitivity encoding factor = 1.5; TE = 6–7 ms; and TR = 10–11 ms. During the acquisition of PC-MRI images, a finger plethysmograph was used for retrospective cardiac gating. All the data were reconstructed into 32 PC images representing the velocity variation within an average cardiac cycle.

PC-MRI images were post-processed using an in-house software, ‘Flow’.^28^ This software implements a semi-automatic segmentation algorithm for blood vessel delineation.

For each segmented pixel within the region of interest (ROI), 32 fluid velocity values were calculated across the cardiac cycle. These values were then averaged and multiplied by the ROI area to compute the total dynamic flow rate through the ROI over the entire cardiac cycle. The software extracted the cerebral arterial inflow as positive values.

The software includes a de-aliasing correction function^28^ for instances where the arterial velocity exceeded the VENC parameter. All post-processing was performed by the same operator (K.O.). A detailed description of the PC-MRI image processing workflow is provided in previous studies,^28,29^ which also validated the inter-rater and intra-rater reliability of the flow measurements. The use of a semi-automatic segmentation algorithm minimizes operator-dependent variability and ensures consistent ROI placement across subjects.

### Lumbar infusion test

A lumbar infusion test was performed in all patients. The procedure was carried out with each patient in the lateral decubitus position, under sterile conditions and using hypnosis techniques to minimize discomfort. An 18-gauge needle was inserted into the lumbar intrathecal space and connected to a pressure transducer and syringe pump via a three-way tap. ICP was continuously recorded using ICM+® software (version 9.1.0.17, https://icmplus.neurosurg.cam.ac.uk/). Baseline ICP was measured over a period of 15–20 min. Subsequently, sterile saline was infused at a constant rate of 1.5 mL/min until a steady-state plateau in ICP was achieved, after which the infusion was stopped. The procedure was performed by a senior neurosurgeon (C.C.), and all patients were closely monitored throughout the test. Upon completion, the ICM+® software automatically calculated the resistance to CSF outflow (*Rout*) as the ratio between the increase in mean ICP (plateau minus baseline) and the corresponding infusion rate, expressed in mmHg/ml/min.^30,31^

### Arterial-related craniospinal physiological compliance

To calculate the arterial-related craniospinal physiological compliance (*C_physio_*), we propose a method based on measurements taken over a single cardiac cycle. The change in cerebral arterial volume (*ΔV_ART_*) is derived from PC-MRI acquisitions, while the corresponding change in ICP (*ΔP_CC_*) is obtained from the lumbar infusion test. The methods used to obtain these values are detailed below.

### ΔV_ART_: cerebral arterial volume change

The total cerebral arterial volume change value during one cardiac cycle (*ΔV_ART_*) was calculated from PC-MRI acquisitions. Total cerebral arterial flow (Q_ART_) was obtained by adding the positive flow curves from the ICAL, ICAR and BA. The cerebral arterial volume change curve (V_ART_) over 32 temporal points across the cardiac cycle was then obtained by integrating Q_ART_ over time. Finally, *ΔV_ART_* was defined as the amplitude of the V_ART_ curve, corresponding to the difference between its maximum and minimum values (Fig. 1B).

### ΔP_CC_: intracranial pressure change

**Figure 2:**
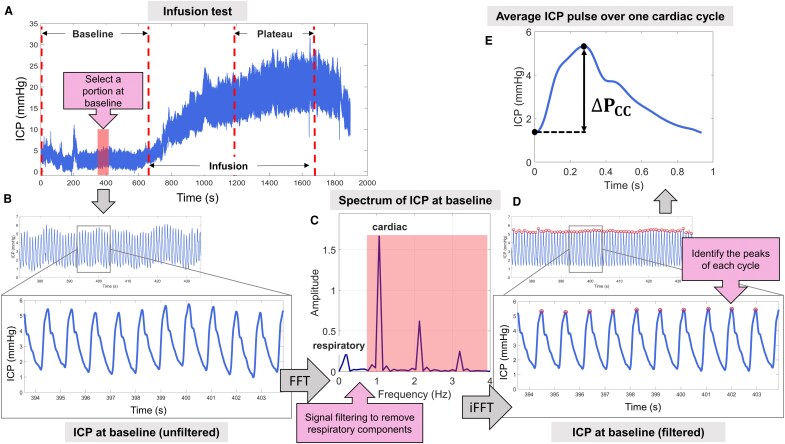
**Workflow for *ΔP_CC_* calculation.** (**A**) The complete ICP signal was exported to in-house software. (**B**) A segment of the baseline ICP, recorded prior to the start of the infusion, was selected from the full ICP signal obtained during the lumbar infusion test. (**C**) The frequency spectrum of this segment was computed using a FFT to filter out noise and respiratory components. (**D**) The cleaned signal was then reconstructed via iFFT. Subsequently, peaks were identified to segment individual ICP pulses. (**E**) Finally, an average ICP pulse over one cardiac cycle was extracted, and the amplitude of the resulted ICP pulse waveform was defined as *ΔP_CC_*.

After insertion of the needle in the lumbar canal, a brief waiting period of a few minutes was allowed to stabilize ICP, reduce patient stress and ensure a high-quality baseline ICP signal. The complete ICP signal was exported to in-house software (Fig. 2A), which was used to segment a few seconds of baseline ICP recorded shortly before the start of the infusion (Fig. 2B). This approach was chosen to ensure comparable physiological conditions to those during the MRI acquisitions—that is, without any stress on the craniospinal system induced by the infusion of artificial CSF. Fourier transform (FFT) was applied to filter out interference from noise and respiratory-related frequencies, followed by an inverse Fourier transform (iFFT) to reconstruct the temporal signal (Fig. 2C). Peaks were identified to segment ICP pulses (Fig. 2D), and the average representative ICP pulse waveform for a single cardiac cycle was obtained (Fig. 2E). Finally, the amplitude of the ICP pulse waveform—defined as the difference between its maximum and minimum values— was defined as *ΔP_CC_*.

Arterial-related physiological compliance was calculated for each patient using the formula:


(1) $$C_{\;physio}\;=\Delta V_{ART}/\Delta P_{CC}$$

### Craniospinal infusion compliance

**Figure 3:**
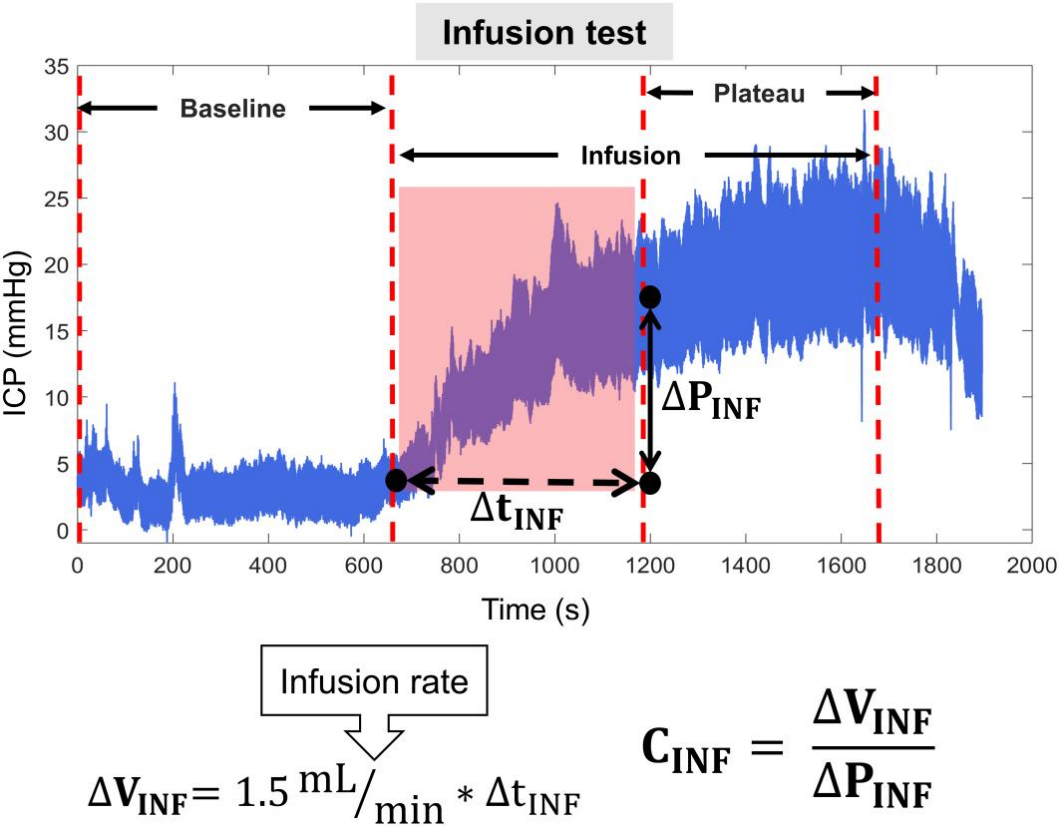
**Craniospinal infusion compliance calculation.** A segment of the ICP signal was selected from the complete ICP recording, spanning from the onset of saline infusion into the spinal subarachnoid space to the point at which the ICP plateau was reached. The change in volume (*ΔV_INF_*) was calculated by multiplying the constant infusion rate (1.5 mL/min) by the duration of the selected segment (*Δt_INF_*). The change in pressure (*ΔP_INF_*) was determined by subtracting the mean baseline ICP value from the mean plateau ICP value. Finally, craniospinal infusion compliance (*C_INF_*) was calculated as the ratio *ΔV_INF_*/*ΔP_INF_*.

To calculate craniospinal infusion compliance-related parameter (*C_INF_*), we analysed ICP recordings obtained during the lumbar infusion test. Specifically, we focused on the segment of the ICP curve between the start of the infusion—when saline solution was introduced into the spinal subarachnoid space—and the point at which the ICP plateau was reached. The change in volume (*ΔV_INF_*) was calculated by multiplying the constant infusion rate (1.5 mL/min) by the duration of the selected segment (*Δt_INF_*). The change in pressure (*ΔP_INF_*) was determined by subtracting the mean baseline ICP from the mean plateau ICP (Fig. 3).

Craniospinal infusion compliance-related parameter was then calculated for each patient using the formula:


(2) $$C_{INF}\;=\Delta V_{INF}/\Delta P_{INF}$$

### Statistical analysis

The statistical analyses were performed with MATLAB software scripts (version 2022a, MathWorks, Natick, WA, USA), and statistical plotting was conducted in Origin (version 2022). The Shapiro–Wilk test was applied to assess the normality of the data. Data are presented as mean ± standard deviation (SD) and illustrated using Q1–Q3 values to provide a comprehensive view of the distribution. The percentage of the coefficient of variation (CV%) was calculated by dividing the standard deviation by the mean and multiplying the result by 100.

Differences between measurements —specifically between heart rates (HRs) during MRI and ICP acquisitions, as well as between volume, pressure, time changes and compliance values—were assessed using either a paired Student’s *t*-test or Wilcoxon’s test, depending on the normality of the data distribution. To account for multiple comparisons across outcome measures, *P*-values were adjusted using the Holm correction. Correlations between the different parameters were assessed using Spearman’s rank correlation test. A significance level of *P* < 0.05 was considered statistically significant for all tests.

## Results

Tables 1 and 2 summarize the results obtained for the entire study population (*n* = 108) and for the subgroup of patients who showed clinical improvement following shunt surgery after being diagnosed with NPH and consenting to the procedure (NPH+, *n* = 29).

**Table 1:** Main parameters derived from PC-MRI and the infusion test in the full cohort (*n* = 108) and in the subgroup of patients with clinical improvement after shunting (NPH+, *n* = 29)

Parameters	Total (*n* = 108)		NPH + (*n* = 29)	
Phase-contrast MRI	mean ± SD [Q1–Q3]	CV (%)	mean ± SD [Q1–Q3]	CV (%)
Heart rate (BPM)	74 ± 14 [64–82]	19	75 ± 15 [63–84]	20
Cerebral arterial mean flow (ml/min)	426 ± 102 [361–481]	24	441 ± 112 [370–483]	25

Infusion test	mean ± SD	CV (%)	mean ± SD	CV (%)
Heart rate (BPM)	61 ± 12 [54–68]	20	63 ± 13 [54–73]	21
Mean ICP baseline (mmHg)	8.52 ± 3.53 [6.11–10.67]	42	9.41 ± 3.01 [7.56–10.99]	32
Mean ICP plateau (mmHg)	28.4 ± 10.3 [21.7–34.6]	36	31.8 ± 10.9 [23.6–33.6]	34
Rout (mmHg/ml/min)	15.7 ± 9.8 [9.6–19.1]	63	20.2 ± 13.7 [12.8–20.6]	68

Data are presented as mean ± SD and [Q1–Q3].

CV, coefficient of variation expressed as percentages; ICP, intracranial pressure; Rout, resistance to CSF flow; SD, standard deviation.

**Table 2:** Physiological and infusion-derived craniospinal compliances in the full cohort (*n* = 108) and in the subgroup of patients with clinical improvement after shunting (NPH+, *n* = 29)

	*ΔV_ART_*	*ΔP_CC_*	*C_physio_*	ΔV_INF_	ΔP_INF_	C_INF_
	(mL)	(mmHg)	(mL/mmHg)	(mL)	(mmHg)	(mL/mmHg)
Total (*n* = 108)						
Mean ± SD [Q1–Q3]	6.0 ± 2.0 [4.6–6.9]	3.0 ± 1.5 [1.8–4.2]	2.7 ± 1.8 [1.4–3.5]	17 ± 6 [14–21]	20 ± 9 [14–19]	1.0 ± 0.5 [0.6–1.4]
CV%	33	50	69	36	45	49
NPH + (*n* = 29)						
Mean ± SD [Q1–Q3]	6.3 ± 2.4 [4.6–7.1]	3.2 ± 1.5 [2.1–4.3]	2.3 ± 1.2 [1.5–2.9]	17 ± 7 [14–19]	22 ± 10 [17–26]	0.9 ± 0.6 [0.6–1.1]
CV%	39	45	53	41	44	62

Data are presented as mean ± SD and [Q1–Q3].

CV, coefficient of variation expressed as percentages; SD, standard deviation.

The mean cerebral arterial flow obtained from PC-MRI is presented in Table 1. Parameters derived from the infusion test are also shown, including the mean ICP at baseline and at plateau, along with the *Rout*. HRs recorded during MRI and lumbar infusion test acquisitions are also reported. A significant difference between the two HR measurements was found in both the full cohort and the NPH + subgroup (paired Wilcoxon’s test, *P* < 0.001).

Table 2 summarizes the results of the physiological compliance (*C_physio_*) measurements along with the infusion-based compliance (*C_INF_*). The corresponding *ΔV* and *ΔP* values used for each craniospinal compliance calculation are also presented.

**Figure 4:**
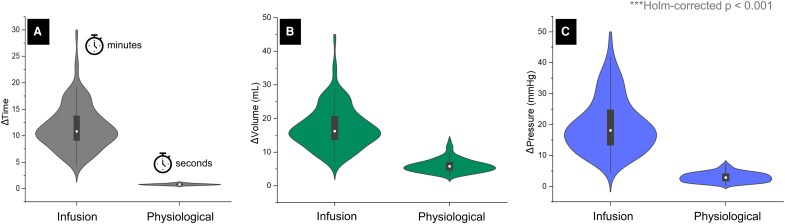
**Comparison of physiological and infusion-derived parameters.** Violin plots show the distributions of (**A**) measurement duration, (**B**) volume change and (**C**) pressure change under infusion and physiological conditions in the full cohort (*n* = 108). For infusion data, time is expressed in minutes, whereas for physiological data, time is expressed in seconds. Boxes within the violins indicate interquartile ranges, with the white dot representing the median. Differences between duration, volume change and pressure change were assessed using paired Wilcoxon’s tests with Holm correction for multiple comparisons (***Holm-corrected *P* < 0.001).

As shown in Fig. 4, craniospinal physiological and infusion compliances are calculated under markedly different conditions. Under physiological conditions, measurements are based on a single cardiac cycle with a duration of 0.83 ± 0.15 s, whereas under infusion conditions, the measurement period is significantly longer, averaging 12 ± 4 min (*P* < 0.001). Additionally, the volume conditions differ markedly: physiological compliance is based on the total cerebral arterial volume change during a single cardiac cycle, measured by PC-MRI, while infusion compliance is derived from the externally administered volume required to stress the system during the infusion test (*ΔV_ART_*: 6.0 ± 2.0 mL versus *ΔV_INF_*: 17 ± 6 mL; *P* < 0.001). Consequently, the associated changes in craniospinal pressure also differ significantly between the two conditions (*ΔP_CC_*: 3.0 ± 1.5 mmHg versus *ΔP_INF_*: 20 ± 9 mmHg; *P* < 0.001). All *P*-values are Holm corrected for multiple comparisons.

**Figure 5:**
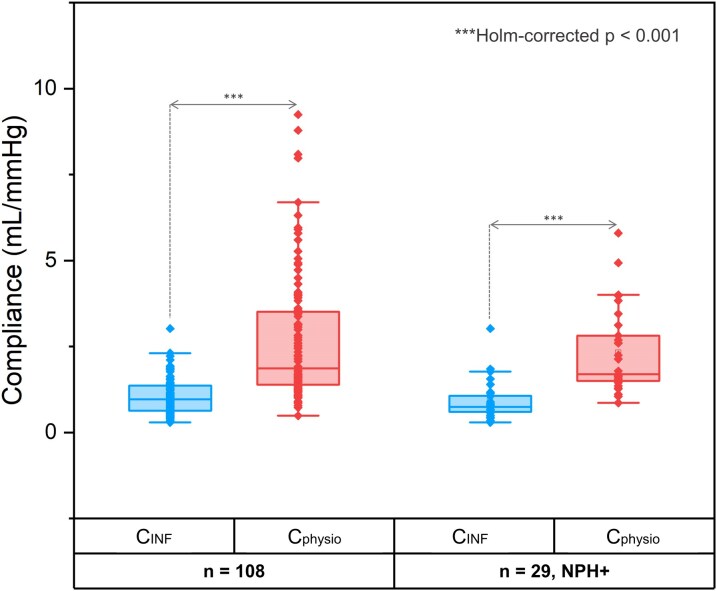
**Distribution of physiological and infusion-derived compliance values.** Boxplots illustrate the distribution of *C_physio_* and C*_INF_* for the full study population (*n* = 108, left) and the NPH + subgroup (*n* = 29, right). In both groups, *C_INF_* values were significantly lower than *C_physio_* measures. Differences between paired compliance measurements were assessed using Wilcoxon signed-rank tests, and *P*-values were adjusted using the Holm correction for multiple comparisons (***Holm-corrected *P* < 0.001).

Figure 5 illustrates the distribution of *C_physio_* and *C_INF_* values for the full cohort (*C_physio_*: 2.7 ± 1.8 mL/mmHg versus *C_INF_*: 1.0 ± 0.5 mL/mmHg) and the NPH + subgroup (*C_physio_*: 2.3 ± 1.2 mL/mmHg versus *C_INF_*: 0.9 ± 0.6 mL/mmHg). In both groups, *C_INF_* values were significantly lower than *C_physio_* values (Holm-corrected *P* < 0.001).

**Figure 6:**
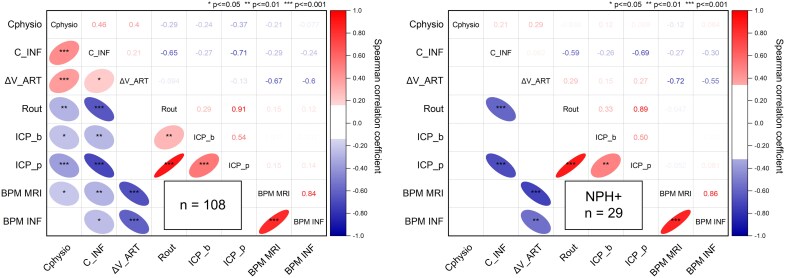
**Correlation analyses among PC-MRI and infusion test parameters.** Spearman correlation matrices: the upper triangle shows Spearman’s rank correlation coefficient (*R*), and the lower triangle displays trend ellipses only where statistically significant correlations were found. Asterisks indicate levels of significance: *P < 0.05; **P < 0.01; ***P < 0.001. *Cphysio* represent the craniospinal physiological compliance (*C_physio_*), and *C_INF* represents the craniospinal infusion-based compliance (*C_INF_*). *Rout* denotes the resistance to CSF outflow; *ICP_b* refers to the mean intracranial pressure during the baseline phase; *ICP_p* refers to the mean intracranial pressure during the plateau phase. *BPM MRI* and *BPM INF* correspond to HRs (in beats per minute) measured during MRI and during the lumbar infusion test, respectively. Left panel: full study population (*n* = 108). Right panel: NPH + subgroup (*n* = 29).

Figure 6 illustrates the correlation matrices between the arterial-related physiological compliance (*Cphysio*), the infusion-derived compliance-related parameter (*C_INF*) and complementary parameters obtained from the lumbar infusion test and MRI acquisitions in the full cohort and for the NPH + subgroup. The analysed parameters include *ΔV_ART_* (*ΔV_ART*), resistance to CSF outflow (*Rout*), baseline mean ICP (*ICP_b*), plateau mean ICP (*ICP_p*) and HR recorded during MRI and during the infusion test (*BPM MRI* and *BPM INF*, respectively).

In the full cohort, *Cphysio* and *C_INF* showed a moderate but statistically significant positive correlation (*R* = 0.46; *P* < 0.001). Both *Cphysio* and *C_INF* showed significant correlations with *Rout* and *ICP_p*, which represented the strongest correlations observed for the compliance parameters, except for the expected association between *ΔV_ART* and *Cphysio*. Within the NPH + subgroup, only *C_INF* remained significantly correlated with *Rout* (*R* = −0.59; *P* < 0.001) and with *ICP_p* (*R* = −0.69; *P* < 0.001).

As complementary findings, significant moderate correlations were also observed between *ICP_b* and *ICP_p* in both the full cohort (*R* = 0.54; *P* < 0.001) and the NPH + subgroup (*R* = 0.50; *P* < 0.01). Additionally, very strong and significant correlations were found between the *ICP_p* and *Rout* in the full cohort (*R* = 0.91; *P* < 0.001) and in the NPH + subgroup (*R* = 0.89; *P* < 0.001). HRs, *BPM MRI* and *BPM INF* were also very strongly correlated in both the full cohort (*R* = 0.84; *P* < 0.001) and the NPH + subgroup (*R* = 0.86; *P* < 0.001). Finally, *ΔV_ART* was strongly and negatively correlated with both *BPM_MRI* and *BPM_INF* in the full cohort and in the NPH + subgroup.

## Discussion

Although compliance is a physical concept with a straightforward mathematical definition (ΔV/ΔP), its application in craniospinal physiology remains complex and not fully understood. A key challenge lies in the fact that craniospinal compliance is not a singular, fixed property—unlike, for example, blood viscosity—but rather an emergent phenomenon resulting from the continuous interplay of dynamic neurofluid flows. Moreover, compliance is highly context dependent and can vary significantly depending on the volume, time and pressure conditions considered. These variations reflect differences in how compliance is defined and measured, rather than implying distinct systems, since a single craniospinal system can exhibit different manifestations of compliance under varying conditions.

The present study aimed, first, to propose a novel estimation—referred to as arterial-related craniospinal physiological compliance (*C_physio_*) —which represents the volume–pressure relationship driven by natural oscillations of arterial blood during the cardiac cycle. Second, we sought to compare the proposed craniospinal physiological compliance with craniospinal infusion-based compliance-related parameter (*C_INF_*), estimated during a diagnostic test in which the system is externally stressed by a controlled volume injection under ICP recording.

It is important to clarify that what is commonly referred to as ICP in this context actually corresponds to the CSF pressure within the craniospinal system measured at the lumbar level, as the term ‘intracranial pressure’ could theoretically encompass different intracranial compartments (e.g. intraparenchymal or intradural). Several studies^32-34^ have demonstrated that mean ICP measured intracranially agrees well with mean ICP measured at the lumbar level. Other studies^35,36^ have reported discrepancies between the two measurement sites, but these differences may arise from the fact that intracranial ICP is sometimes measured intraparenchymally rather than intraventricularly, which does not represent the same fluid compartment as lumbar ICP pressure. Furthermore, although a phase shift in ICP may exist between the intracranial and spinal compartments, this should not have influenced the compliance estimates, as the same analytical approach was consistently applied across all patients and measurements.

In our centre, we are fortunate to systematically apply PC-MRI and lumbar infusion test to all patients evaluated for suspected NPH. Building on this advantage, we combined the arterial volume change (*ΔV_ART_*) obtained from PC-MRI with the ICP change (*ΔP_CC_*) measured at baseline from the infusion test (prior to infusion), both calculated over one cardiac cycle, in order to maintain the most comparable physiological condition possible between the two modalities. This approach enabled us to propose a novel parameter for estimating an arterial-related craniospinal physiological compliance.

### Phase-contrast MRI and infusion test-derived parameters

From PC-MRI, the total mean cerebral arterial flow was 426 ± 102 mL/min for the full cohort of suspected NPH patients. This value appears significantly reduced compared with those reported for healthy elderly populations in the literature.^19,37,38^ El Sankari *et al.*^39^ reported a mean cerebral arterial flow of 450 ± 129 mL/min in NPH patients, comparable to our finding, while Bateman *et al.*^13^ observed slightly higher values, 570 ± 150 mL/min, in NPH responders but noted an ∼20% reduction compared with their normal elderly group. Other studies using different imaging techniques, such as PET^40^ and computed tomography perfusion (CTP),^41^ have also reported global reductions in cerebral blood flow in NPH patients.

Compared to the findings of Jacobsson *et al.*,^42^ who reported a baseline ICP pulse amplitude of 2.4 ± 1.3 mmHg in a cohort of 62 NPH patients, our result for *ΔP_CC_* was slightly higher, 3.0 ± 1.5 mmHg.

Although HR differed significantly between the MRI and infusion test conditions (Table 1), a strong positive correlation was observed between the two measurements (Fig. 6). This indicates that individuals with higher HR during MRI also tended to have higher HR during the infusion, thereby preserving the relative ranking among subjects. Because HR strongly influences arterial volume changes (Fig. 6), such differences could theoretically affect the estimation of arterial-related physiological compliance. However, given the strong correlation between HR measurements and the consistent methodology applied across all patients, any HR-related bias would likely represent a uniform offset across the cohort rather than a differential effect between individuals. Therefore, the relationships observed in physiological compliances are expected to remain valid.

### Arterial-related craniospinal physiological compliance

Since, in the lumbar infusion test, pressure is measured without any obstruction between compartments, the resulting ICP reflects the dynamics of the entire craniospinal system. Therefore, in this study, the goal was to assess craniospinal compliance. To ensure comparability with infusion-based compliance, only the cerebral arterial volume input was considered in the estimation of physiological compliance. Conversely, if the objective were to estimate intracranial compliance specifically, both blood and CSF volume changes should be included, whereas spinal compliance would be derived primarily from CSF volume changes.

The observed statistically significant correlations between *C_physio_* and a subset of physiological and infusion-derived parameters in the full cohort (Fig. 6) highlight the complexity of compliance as a multifactorial phenomenon. Interestingly, none of these correlations were statistically significant within the NPH + subgroup. This could partly be attributed to the smaller sample size (*n* = 29), which may limit statistical power to detect modest associations. Moreover, it is possible that the lack of significant correlations may also reflect the heterogeneous nature of NPH pathophysiology, which could introduce interindividual variability in compensatory mechanisms or anatomical alterations, thereby confounding systematic associations within the NPH + group.

It is important to note that the NPH + subgroup represents only the subset with complete pre- and post-shunt data confirming clinical improvement—not the total number of improved or operated patients—therefore the full cohort remains critical for the analysis. In fact, evaluating the entire group enhances our ability to uncover moderate associations that might not be evident within smaller subgroups. Moreover, all 108 patients included in the study underwent both MRI and invasive lumbar infusion testing based on strong clinical suspicion of NPH, further supporting the relevance of the full cohort in exploring the relationships between physiological and clinical parameters.

Alperin *et al*.^11^ proposed a non-invasive MRI method that estimates intracranial compliance from PC-MRI–derived volume changes and CSF pressure gradients. However, their model assumes rigid conduits and uniform CSF flow along the spinal axis—assumptions that depart from physiological reality, as demonstrated by Sass *et al*.^43^ Although widely used in NPH populations, this approach has shown limited agreement with invasive ICP measurements.^44^ In contrast, our approach integrates directly measured lumbar pressure with PC-MRI data, providing a more physiologically realistic compliance estimate.

To our knowledge, only Wåhlin *et al*.^9^ previously combined PC-MRI and the lumbar infusion test to estimate craniospinal compliance using pressure–volume indices (PVIs), defined as the volume required to increase ICP 10-fold.^45^ Their analysis was based on mean ICP and ΔP relationships obtained throughout the entire infusion test, assuming that the pressure increase induced by saline infusion does not substantially alter neurofluid dynamics. In contrast, our approach estimates compliance from baseline ICP data, reflecting physiological, unstressed conditions. Moreover, although both methods rely on PC-MRI–derived cerebral arterial volume change, a key methodological difference lies in how *ΔV_ART_* is computed. We calculated the total arterial volume variation over one cardiac cycle (without subtracting the mean flow), whereas Wåhlin *et al*. considered only the pulsatile arterial volume component after removal of the mean flow. Importantly, these two approaches address different, yet complementary, physiological questions. The approach proposed by Wåhlin *et al*. focuses on isolating arterial wall expansion and compression, thereby specifically examining how arterial pulsatility contributes to the generation of the ICP pulse. In contrast, our approach aims to characterize how the arterial volumetric input to the craniospinal system over the cardiac cycle relates to the resulting ICP pulsation, thereby reflecting the effective arterial-related pressure–volume response of the system. Within a mathematical ΔV/ΔP framework, both definitions are valid, but they emphasize different aspects of the volume change considered and, consequently, different physiological interpretations of compliance.

In addition, we measured cerebral arterial flow at the intracranial level, whereas Wåhlin *et al*. acquired it extracranially. Although arterial variability between these levels is relatively small compared with venous variability,^46^ intracranial acquisition ensures better temporal synchronization with the CSF response at the C2–C3 level,^47^ which represents an advantage if venous or CSF compartments are to be incorporated in future analyses. Finally, our study was conducted in patients with suspected NPH, whereas Wåhlin *et al*. investigated healthy elderly subjects, further highlighting the novelty and clinical relevance of the present work.

In the present study, physiological craniospinal compliance was estimated using cerebral arterial volume change alone. However, assessing CSF and venous flows remains of great physiological relevance, as these compartments capture complementary aspects of the craniospinal system’s compensatory behaviour. Depending on which volume change is considered—arterial, venous, or CSF—each approach explores a distinct component of craniospinal compliance. Future studies combining these complementary measurements may provide a more comprehensive understanding of the integrated compliance mechanisms governing craniospinal dynamics.

To our knowledge, this is the first study to combine the lumbar infusion test and PC-MRI under comparable physiological conditions to provide a quantitative estimation of arterial-related craniospinal physiological compliance.

### Comparison of craniospinal infusion-based and physiological compliance

As shown in Fig. 4, the volume, pressure and time scales used for the calculation of *C_physio_* and *C_INF_* differ significantly (*P* < 0.001), leading to fundamentally different measures of craniospinal compliance. Specifically, the infusion test involves approximately three times higher volume, six times higher pressure and 841 times longer duration compared to the physiological conditions used to compute *C_physio_*. These differences arise from the nature of the two measurement approaches.

In this study, we intentionally adopted a simple operational definition of compliance (C=ΔV/ΔP) and compared the two approaches empirically using only measured volume and pressure changes. Although the physiological mechanisms differ between the two conditions, this direct comparison provides insight into how the craniospinal system responds across distinct temporal and volumetric scales.

*C_INF_* reflects the integrated response of the craniospinal system under stress conditions induced by the external infusion of artificial CSF over several minutes, resulting in relatively large and sustained changes in pressure and volume. It involves compensatory mechanisms traditionally described by the Monro–Kellie doctrine, which states that the total intracranial volume—comprising brain tissue, blood and CSF— is constant and confined within a rigid, closed cranial vault, under the assumption that these compartments are incompressible.^48^ Among the compensatory responses triggered by infusion-induced increases in ICP, several studies have demonstrated that elevated pressure significantly enhances the rate of CSF resorption, whereas CSF production appears to remain relatively stable and largely insensitive to changes in ICP.^49,50^ Additional compensatory mechanisms may include slight downward displacement of the cerebellar tonsils through the foramen magnum^51,52^ and redistribution of intradural CSF into the spinal canal. Furthermore, reductions in intracranial arterial, capillary or venous blood volumes may occur, along with compression of intradural brain tissue. These mechanisms reflect an effort to maintain volume equilibrium within a closed system.

However, this traditional view has increasingly been challenged.^53,54^ Certain anatomical openings, such as the orbits and tympanic cavities, allow partial pressure interaction with adjacent compartments, suggesting that the intradural volume is not perfectly constant. Moreover, the skull has been shown to undergo shape changes in adults with intracranial hypotension or intracranial hypertension, indicating that the cranial vault is not entirely rigid.^55^

For instance, elevated CSF pressure has been shown to cause flattening of the globe in cases of intracranial hypertension,^56^ while chronic intracranial hypotension—such as in over-shunted patients—has been associated with orbital expansion.^57^ Additionally, clinical studies increasingly support the association between millimetric increases in the sonographic optic nerve sheath diameter (ONSD) and raised ICP.^58^ Hansen *et al.*^59^ demonstrated that during a lumbar intrathecal infusion test, ONSD dilation reaches a maximum at peak CSF pressure. In parallel, several studies^60,61^ have shown that ICP fluctuations can also affect tympanic membrane displacement (TMD), which is measured as changes in ear canal volume over time. Other research studies^62,63^ have explored non-invasive ICP monitoring via TMD, a technique based on the presumed anatomical connection between the intracranial subarachnoid space and the perilymphatic space of the inner ear via the cochlear aqueduct.

In contrast, *C_physio_* captures the rapid, small-volume fluctuations of the craniospinal system that occur under physiological conditions during each cardiac cycle. Over this short time scale, compensatory mechanisms are limited, and transient changes in volume and pressure arise from the pulsatile arterial inflow and redistribution of venous blood and CSF. When such rapid variations in intracranial volume occur, the assumptions of the Monro–Kellie doctrine no longer fully apply. Several studies^47,64,65^ have shown that, particularly over the time scale of a cardiac cycle, changes in total intracranial volume do occur, further questioning the assumption of volume constancy. These observations support the notion of an ε-compressibility—a small but non-negligible compressibility—or an ε-intradural volume change within cranial and spinal compartments, which contributes to the overall craniospinal compliance. Under these short-term dynamic conditions, ICP regulation is achieved primarily by the pulsatile displacement of CSF between the intracranial and spinal subarachnoid spaces, which buffers the rapid intracranial arterial volume increase during systole. In contrast, venous compensation becomes more relevant for slower or sustained changes in intracranial volume.^53^

To conclude, our results showed that physiological (cardiac-cycle–based) and infusion-based craniospinal compliances, although different, are moderately correlated and therefore complementary. Both reflect the behaviour of a single craniospinal system but capture its distinct capacity to respond under different volume, time and pressure conditions. By evaluating both approaches within the same patient population, this study highlights the need to consider the context-dependent nature of craniospinal compliance and the limitations of relying on a single measurement method to capture the full dynamic behaviour of the craniospinal system. A better understanding of this complexity may contribute to the development of more precise diagnostic tools and better-targeted interventions for patients with suspected NPH.

### Limitations

It is important to highlight that the lumbar infusion test and PC-MRI were not performed simultaneously. However, we deliberately ensured that MRI acquisition preceded the infusion test, as the latter intentionally disturbs the craniospinal system’s dynamics through the injection of saline into the spinal subarachnoid space. To minimize temporal variability, we included only patients who underwent both examinations within a maximum interval of 5 months, with the majority of patients completing both tests within a few weeks. Importantly, both examinations were performed under stable clinical conditions, with no major clinical events or therapeutic changes occurring between the two procedures.

Body posture also differed between the two procedures (lateral decubitus position during the infusion test and supine position during MRI acquisition). Nevertheless, as stated by Norager *et al*.,^66^ differences in lumbar ICP measurements between the lateral recumbent and supine positions have little clinical relevance. Moreover, in both postures, the influence of gravity is minimized, whereas gravitational effects have been demonstrated to be one of the main factors affecting ICP^67,68^ and neurofluid dynamics.^69,70^

Additionally, performing the infusion test in an MRI-compatible setting to allow simultaneous MRI acquisition remains ethically unacceptable due to safety concerns and patient discomfort and is also technically challenging. Some studies^11,12,71^ have attempted to estimate ICP and compliance using MRI-based measurements alone; however, this approach has proven to be a poor surrogate marker for ICP change in NPH patients.^44^

As the lumbar infusion test and PC-MRI were not performed simultaneously, patients’ HRs were not identical during the two procedures. Indeed, we observed a significantly lower HR during the lumbar infusion test (Table 1), which is expected since the procedure is conducted under hypnosis techniques designed to relax the patient and thereby reduce HR. Nevertheless, as shown in Fig. 6, a strong correlation was observed between the HRs measured during MRI and ICP acquisitions.

### Phase-contract MRI acquisitions

While ICP can be measured with relatively high precision, such measurements remain invasive, uncomfortable and ethically difficult to justify in the absence of a clinical indication. In contrast, PC-MRI offers a non-invasive alternative that can be safely applied to healthy individuals. Nonetheless, it is not without some limitations.

The choice of VENC must be tailored to each fluid compartment: setting the VENC too high reduces sensitivity to low-velocity flows, whereas setting it too low can result in aliasing artefacts. Additionally, the acquisition plane must be oriented as perpendicular as possible to the direction of flow to avoid underestimating velocities. In our protocol, careful attention was paid to selecting appropriate VENC values and ensuring optimal plane orientation across all acquisitions.

The presence of air produces a high susceptibility to artefacts, particularly in regions close to the paranasal sinuses, where significant field inhomogeneities can distort the image.^72^ Acquisitions at the intracranial level were obtained close to the nasal cavity, which could produce high susceptibility to image artefacts indeed. However, in this study, the artefacts caused by air were relatively minor, and their impact on the accuracy of the blood flow measurements was minimal.

It has been shown that not only cardiac activity but also respiration contributes to cerebral blood and CSF volume changes.^73-75^ However, standard cardiac-gated PC-MRI acquisitions do not capture the influence of respiration on neurofluid volume fluctuations. As a result, any compliance changes related to respiratory-induced dynamics remain unaccounted for in this study. Investigating breathing-related compliance could be an important area for future research, potentially using alternative MRI techniques, such as real-time phase-contrast (RT-PC) MRI, which allow simultaneous acquisition of respiratory and cardiac components. Nevertheless, RT-PC MRI still presents some limitations, including lower spatial resolution and signal-to-noise ratio compared to conventional 2D cine PC-MRI, which may reduce the accuracy of small volume change quantification.

### Intracranial pressure acquisition

Several studies suggest that total CSF compliance can be subdivided into cranial and spinal components. Marmarou *et al.,*^30^ estimated that approximately two-thirds of the total CSF compliance resides within the cranial compartment in anaesthetized adult cats with craniospinal compartment isolated at c6 level. Magnæs,^76^ in turn, investigated human patients with CSF block at the cervical level and demonstrated that the relative contribution of each compartment depends on body position. In the horizontal position, the cranial compartment contributed 37% and the spinal compartment 63% to the total compliance of the craniospinal system. In the erect position, the proportions were nearly reversed, with 66% cranial and 34% spinal contributions. The total compliance was almost unaffected by body position. More recent work by Burman *et al.,*^77^ using a lumped-parameter model combined with non-invasive ICP estimation from PC-MRI, found that the spinal canal contributed the majority of compliance in subjects up to 40 years of age. However, in the 41–60-year age group, no single sub-compartment clearly dominated.

In our study, ICP was measured in the spinal compartment without any artificial blockade. Under these conditions, the average values of spinal and ICPs can be considered equivalent—apart from a physiological time delay—since a pressure gradient is required to facilitate CSF flow through the craniospinal axis. Therefore, the ICP waveform recorded during the lumbar infusion test reflects the neurofluid volume change occurring over a single cardiac cycle, along with the compliance of the entire craniospinal system. While this approach is appropriate for assessing craniospinal compliance, it may not isolate intracranial compliance specifically. Therefore, comparisons with studies focused solely on intracranial dynamics should be made with caution.

## Data Availability

DICOM data cannot be provided due to privacy laws. De-identified processed data can be provided upon reasonable request. To obtain the post-processing software and its user manual, please contact Dr. Olivier Balédent.
